# Study of Interatomic Potentials in ZnS—Crystal-GRID Experiments Versus Ab Initio Calculations

**DOI:** 10.6028/jres.105.010

**Published:** 2000-02-01

**Authors:** Timo Koch, Karl-Heinz Heinig, Michael Jentschel, Hans G. Börner

**Affiliations:** Forschungszentrum Rossendorf, D-01314 Dresden, Germany; Institut Laue-Langevin, F-38000 Grenoble, France; Forschungszentrum Rossendorf, D-01314 Dresden, Germany; Institut Laue-Langevin, F-38000 Grenoble, France; Institut Laue-Langevin, F-38000 Grenoble, France

**Keywords:** atomic collisions, Crystal-GRID, gamma ray spectroscopy, inter-atomic potentials, Molecular Dynamics simulations, nuclear lifetimes, ZnS

## Abstract

Crystal-GRID measurements have been performed with ZnS single crystals. For the first time, an asymmetric Crystal-GRID line shape could be observed. The preliminary data evaluation indicates that the reported lifetime of the 3221 keV level in ^33^S is too short. A value of about 60 fs has been found. Due to this “long” lifetime the line shape is much less structured than calculated with the reported lifetime.

## 1. Introduction

During the last decade the Gamma Ray Induced Doppler broadening (GRID) technique has been optimised at the Institut Laue-Langevin (ILL). It has been successfully applied to lifetime measurements. A description of the method can be found in Ref. [1].

The use of single-crystalline samples for GRID experiments was proposed during the first GRID workshop in 1992 [2]. This special application, called “Crystal-GRID”, should facilitate the study of interatomic solid state potentials, lifetimes of nuclear states, and impurity locations in the lattice. In recent works [3,4,5] the general applicability of the new method to the study of interatomic potentials has been proven.

In this paper a preliminary report on measurements with ZnS single crystals will be presented. These experiments aimed to observe for the first time a theoretically predicted asymmetry [2] of the Doppler broadened line shape for the 〈111〉 crystal direction aligned to the spectrometer axis. It is expected that such an asymmetry allows the determination of interatomic solid state potentials and nuclear lifetimes to a higher precision than with symmetric line shapes.

## 2. The Crystal-GRID Technique

In a thermal neutron capture reaction the binding energy of the neutron is deposited in the nucleus and the deexcitation takes place via a *γ*-cascade. The emission of a first, primary photon *γ*_1_ entails a recoil of the still excited nucleus due to the conservation of momentum *p*:

$$p=mv_{0}=E_{\gamma_{1}}/c,$$ (1)

where *m* is the mass and *v*_0_ the initial velocity of the recoiling nucleus, $E_{\gamma_{1}}$ the energy of the primary photon and *c* the velocity of light. As the energy of the first photon is of the order of several MeV, the initial velocity *v*_0_ of the recoiling atom lies in between 10^−5^ *c* and 2 × 10^−4^ *c*, corresponding to an initial kinetic energy *E*_kin,0_ of approximately 100 eV to 1000 eV. The direction of the initial recoil is arbitrary. The further motion of the recoiling nucleus is defined by the interatomic forces, i.e., by the interatomic potential in the solid.

At the time *t*′ a second photon *γ*_2_ is emitted and observed by the spectrometer if emitted along the direction of observation. If the nucleus [velocity *v*(*t*)] has not yet come to rest, the *γ* energy is Doppler shifted by

$$\Delta E_{\gamma_{2}}=E_{\gamma_{2}}^{0}\frac{v(t^{\prime })\cdot n}{c},$$ (2)

where $E_{\gamma_{2}}^{0}$ is the unshifted energy of the second photon and ***n*** the unit vector in the direction of observation. Due to the total number of Doppler shifted photons a Doppler broadened photon energy spectrum is obtained.

The motion of the recoiling atoms within a single crystal is highly anisotropic. For example, the slowing down in the direction of the nearest neighbour (“blocking”) is more efficient than in the direction between two nearest neighbours (“channeling”). Therefore, the isotropic distribution of velocities just after the recoil becomes anisotropic as soon as the interaction with nearest neighbours becomes non-negligible. In a powder target this anisotropy is averaged out by the arbitrary alignment of the micro-crystals. However, in the case of a single crystal this anisotropy exists macroscopically and leads to a fine structure in the Doppler broadened line shape. As the fine structure is caused by the interaction of the recoiling atom with its neighbours it represents a sensitive indicator for the interatomic potential.

Crystal-GRID measurements can be performed at the high-flux neutron reactor of the Institut Laue-Langevin in Grenoble. The crystals to be investigated are placed in a distance of 55 cm from the reactor core in a tangential beam tube, where they are permanently irradiated by thermal neutrons (≈ 5 × 10^14^ neutrons/cm^2^s). In general, three oriented crystals of size 17 mm × 20 mm × 2 mm are used for the measurements. Two high-resolution gamma spectrometers, GAMS 4 and GAMS 5, are placed on either side of the beam tube at a distance of 13 m and 17 m of the samples, respectively. Both operate in double flat crystal geometry, i.e. energy resolution is gained by successive Bragg reflection on two almost perfect single crystals.

## 3. Requirements of Crystals To Be Studied

Theoretically, Crystal-GRID studies can be performed with a large number of materials, however the sensitivity of the Doppler-broadened line shapes to the atomic interaction depends on a number of criteria. First of all, the material should contain an isotope, which has a simple *γ* cascade with a mostly primarily fed short living (5 fs to 40 fs) level. The primary feeding guarantees an almost monoenergetic recoil energy, the short lifetime the restriction to a few collision processes. Further, the coordination number of the material itself should be as low as possible. In this case the anisotropy of the recoil motion and therefore the fine structure in the Crystal-GRID line shape is strongly pronounced.

In an experiment a number of additional requirements have to be met: Currently, the intensity requirement is the most limiting. For that reason sufficiently big single crystals are needed. The typical size used up to now is three pieces 17 mm × 20 mm × 2 mm. As soon as GAMS 5 is operational in bent-crystal mode, thin single-crystalline films should also be usable. Further, the measured intensity depends on a number of quantities such as the capture cross section, the absolute intensity of the transition, and the response function of the spectrometer, which itself depends on the energy as well as the thickness and the material of the crystals used in the spectrometer. According to the requirement of a simple *γ*-cascade with short lived levels, light nuclei would be preferential. However these isotopes do often have a very small neutron capture cross section. This limits currently the number of candidates for Crystal-GRID experiments to isotopes such as ^36^Cl, ^49^Ti, ^56^Fe, ^59^Ni, ^52^Cr, and ^33^S. Therefore improvements of the spectrometer efficiency are urgently needed. A first step will be done with GAMS 5. An efficiency improved by a factor of 100 would be needed, in order to be able to investigate alot of materials that are of great interest in solid state physics, for example, a number of important semiconductors.

Because the crystal temperature can reach 1200 K in the reactor, the crystal structure needs to be stable up to this temperature region. Furthermore reactor safety requirements must be considered (β-activity, no melting or evaporation of material, etc.).

Once these basic requirements are fulfilled, theoretical calculations can be performed (see Sec. 4). If a line shape is very structured or not, depends mainly on the lifetime and the crystal lattice. Short lifetimes and open structures lead to the most pronounced line shapes.

## 4. Theoretical Description

An analytical calculation of theoretical line shapes is only possible for simple assumptions on the slowing down process. Crystal-GRID line shapes can not be deduced with sufficient accuracy in that way. We have to use computer simulations instead. As the recoil velocities of the atoms are much below typical electron velocities (≈ 7 × 10^−3^ *c*), electronic excitations are not taken into account. This allows consideration of the recoil motion within classical mechanics. The most exact computer simulation approach within classical mechanics are molecular dynamics (MD) calculations. In these calculations the motion of *N* atoms within a finite volume *V* is calculated assuming certain initial and boundary conditions and the knowledge of the atomic interaction of all *N* atoms. The trajectories of the particles can be calculated by simultaneously integrating Newton’s equations of motion of all *N* particles. In the case of recoil simulations for Crystal-GRID experiments the initial conditions are defined by the crystal structure of the targets, where all atoms move at an initial velocity following a Maxwell distribution corresponding to the temperature of the crystal. The simulation volume is a cubic cell, the size of which is adjusted such that the recoiling atoms do not reach the boundaries within the time of simulation. Periodic boundary conditions are used. Starting with an initial recoil, possible trajectories can be simulated. The method is exact within classical mechanics. Hence the result of MD simulations is as good as the interatomic potential. However, MD simulations are very time consuming. For our purpose only the interaction of the recoiling nucleus with all its neighbouring nuclei needs to be known. The interaction of two nuclei far away from the recoiling nucleus has no appreciable influence and can be neglected. This approximation is called “restricted molecular dynamics” (RMD) and is sufficient for Crystal-GRID simulations [3].

For ZnS 1400 trajectories ***r****_i_*(*t*) with velocities ***v****_i_*(*t*) have been simulated with an RMD program. For better statistics the symmetry operations of the appropriate point group can be applied to the trajectories. In order to compare simulation and experiment the set of trajectories needs to be transformed to a probability distribution of velocities or energies. In an actual measurement the direction of observation is fixed. As seen in Eq. (2), the Doppler shift only depends on the projection *v*_‖_ = ***v***·***n*** of the velocity on the direction of observation.

For a given ***n*** the velocity projection distribution *P*(*v*_‖_,*t*) d*v*_‖_ d*t* can be deduced by counting the trajectories having a velocity projection ***v****_i_*(*t*)·***n*** in the range *v*_‖_ … *v*_‖_ + d*v*_‖_ during the time interval *t*…*t* + d*t*. A normalisation of the distribution is not necessary, because the measurement does not yield absolute intensities. This distribution is independent of the lifetime of the nuclear state, because the trajectories have been calculated for a fixed period of time regardless of the second decay.

The distribution *P*(*v*_‖_) d*v*_‖_ of velocity projections *v*_‖_ at the time of the second *γ* decay can then be calculated for a given lifetime *τ* by multiplying the radioactive decay law:

$$P(v_{∥})dv_{∥}=\int_{0}^{5\tau }\exp\left(-\frac{t^{\prime }}{\tau }\right)P(v_{∥},t^{\prime })dt^{\prime }$$ (3)

The integral is calculated up to 5*τ*, where 99.3 % of the secondary photons have been emitted. Using Eq. (2) this can be transformed to the probability distribution $P(E_{\gamma_{2}})$ $\;dE_{\gamma_{2}}$ of the Doppler shifted energy, the so called Crystal-GRID line shape

$$P(E_{\gamma_{2}})\;dE_{\gamma_{2}}\sim P\left(v_{∥}=\frac{c\Delta E_{\gamma_{2}}}{E_{\gamma_{2}}^{0}}\right)\;dE_{\gamma_{2}}$$ (4)

This line shape would be measured with a spectrometer yielding a δ-like response function. In the case of an ideal double flat crystal spectrometer the instrumental response function can be calculated from diffraction theory. Due to small imperfections of the used spectrometer crystals an additional broadening has to be taken into account by folding the ideal instrument response function with a Gaussian of width *σ*_EW_, the so called “excess width”. The later can be determined experimentally. When folding $P(E_{\gamma_{2}})$ with the obtained realistic response function, the line shape to be fitted to the experimental data can be calculated (for details see [1]).

As can be seen, the measured Crystal-GRID line shapes are directly correlated to the slowing down process in the crystal. For a given crystal structure and alignment of the crystal, the calculated line shape depends only on the classical potential used in the RMD calculation and on the level lifetime. Therefore, by comparing simulation and measurement, the interatomic potential may be improved in the energy region of 10 eV to 1000 eV.

## 5. The Zincblende Structure

Under normal conditions, Zinc sulfide (ZnS) has the zincblende structure (point group $F\bar{4}3m$). The two constituents are found on two face-centred cubic sublattices respectively, displaced with respect to each other by one forth of the diagonal of the cubic unit cell. In GRID experiments the direction of observation, i.e., the direction of the axis between the sample and the spectrometer, is well defined.

In previous Crystal-GRID measurements the Doppler broadening of the line shape was always symmetric with respect to the unshifted photon energy. This symmetry was due to the existing inversion symmetry in real space of the crystals. Crystals having an inversion symmetry yield symmetric line shapes for all orientations, because the nucleus slows down in the same way for an emission with velocity +***v***_0_ or −***v***_0_. The GRID method is insensitive to rotations of the sample around the direction of observation. Therefore a symmetric line shape is also produced if the lattice only has a rotation-inversion symmetry around this axis.

The zincblende structure has no inversion symmetry. However a rotation-inversion symmetry exists for some axes, e.g., for the 〈100〉 and the 〈110〉 directions, but not for the 〈111〉 direction. Consequently the expected line shape in the latter case is asymmetric.

## 6. Experiments

**Fig. 1:**
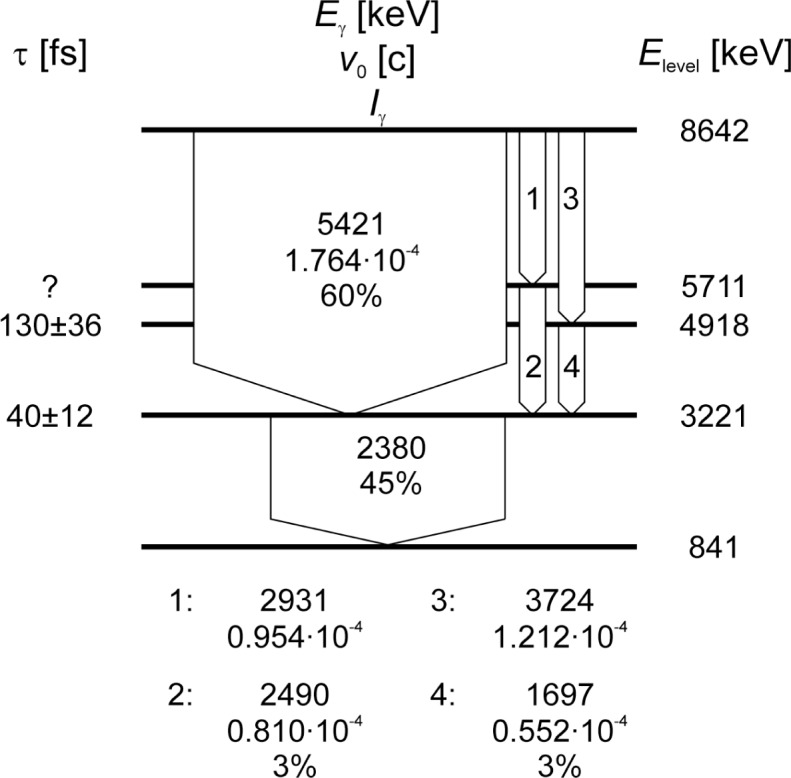
Partial level scheme of ^33^S [6]. For each level of energy *E*_level_ the reported lifetime *τ* is given [7]. For the transitions the photon energy *E_γ_*, the corresponding initial recoil velocity *v*_0_ of the S nucleus, and the absolute intensity of the transition (% means per 100 neutrons) are given.

During two measuring periods we examined the ZnS crystals with the 〈111〉 and 〈110〉 directions respectively oriented towards the spectrometer. The measurements used the 2380 keV transition in the ^33^S nucleus, depopulating the 3221 keV level. This level is mostly directly fed, 91 % of the populating nuclei decay directly from the capture state (see Fig. 1). Two two-step cascades further contribute to the feeding [6]. Their influence is small but taken into account in the RMD simulations. The energy of the first photon is 5421 keV, corresponding to an initial recoil energy of the excited S nucleus of 478 eV, i.e., a recoil velocity of 1.76 · 10^−4^*c* or 0.529 Å/fs. The nuclear state has a reported lifetime of (40 ± 12) fs [7].

The double crystal spectrometer was used with one crystal in first and the other in second reflection order. For each orientation of the crystal 35 scans have been measured. Every scan consisted of 90 data points, each of them being measured for 320 s. Thereby a peak intensity of about 40 counts per scan could be attained.

**Fig. 2:**
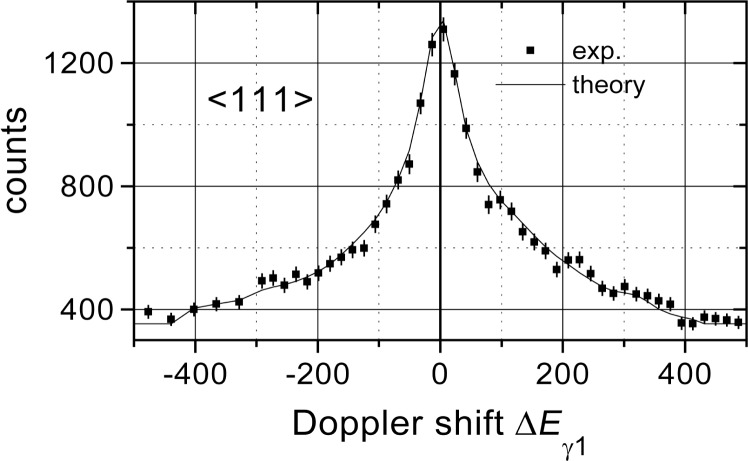
Doppler-broadened line shape of the 2380 keV transition in ^33^S for the 〈111〉 direction. The line represents the fitted theoretical line shape obtained with RMD simulations using the ZBL potential and a level lifetime of 65 fs. The asymmetry can be seen in the wings of the curve around 120 eV and in the small peaks between 200 eV and 300 eV.

**Fig. 3:**
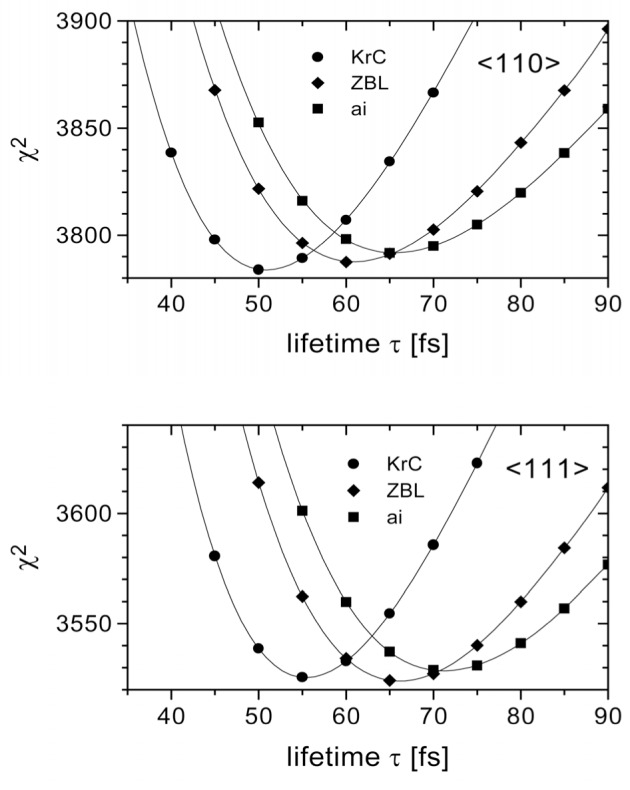
Determination of the lifetime of the 3221 keV level in ^33^S. Computer simulations have been performed for different screened Coulomb potentials (KrC, ZBL). For each crystal orientation theoretical line shapes for different lifetimes can be fitted to the experimental data. The calculated *χ*^2^ values are plotted against the lifetime. The minima of the curves give the resulting lifetimes. They vary around 60 fs. Using the preliminary potential obtained from *ab initio* calculations, a lifetime around 70 fs would be obtained. However the *χ*^2^ values are worse than for the screened Coulomb potentials. The *ab initio* results need to be evaluated more carefully.

The data evaluation is still in progress. Up to now we obtained two preliminary results. On the one hand, for the first time, the predicted asymmetry of the line shape in the 〈111〉 direction could be confirmed (see Fig. 2). On the other hand the lifetime of the nuclear state under investigation was found to be much longer than the reported value [7]. Considering two different interatomic potentials (ZBL [8], KrC [9]) in our theoretical model and fitting the results of the two crystal orientations independently, we obtain a lifetime around 60 fs as can be seen in Fig. 3. The fitted lifetimes for the two different directions are not identical. This indicates that the interatomic potentials used in the simulation do not well describe the interaction. Consequently a new potential will be developed.

Due to the long lifetime the line shapes are much less structured than predicted by the computer simulations performed before the experiment with the reported lifetime. Thus, the information which can be gained for the interatomic potentials will be reduced.

## 7. *Ab Initio* Results

Our second, purely theoretical approach to ZnS potentials consists of quantum mechanical — so called *ab initio* — calculations. These calculations allow to obtain the total energy of an arbitrary configuration of typically 64 atoms without requiring any information about classical interatomic potentials. By calculating the energy of a series of atomic configurations, an interatomic potential can be developed theoretically.

**Fig. 4:**
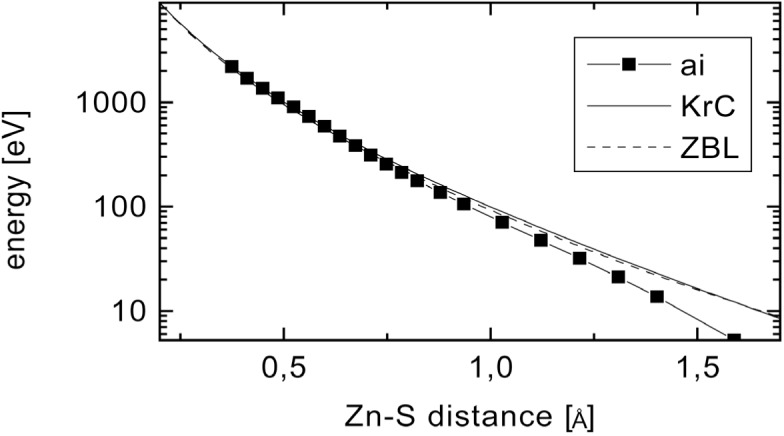
The energy of a 64-atom lattice with one S atom displaced along the 〈111〉 direction towards the nearest neighbour has been *ab initio* calculated. The difference to the energy of the ideal lattice is plotted versus the Zn—S distance. The nearest neighbour distance in the ideal lattice is 2.33 Å. The obtained relation can be seen as an *ab initio* potential and is compared to the classical screened Coulomb potentials used in this work (KrC, ZBL).

In a first step one of the S atoms within a simulation cell of 64 atoms was displaced along the 〈111〉 direction towards its nearest neighbour. For different distances the total energy has been calculated. The difference to the total energy of the ideal lattice gives a first approach to an *ab initio* potential (see Fig. 4). Even though not only the Zn—S interaction between two particles is considered, the contribution of other atom pairs should be comparatively small.

RMD calculations have been performed with this deduced *ab initio* potential. As can be seen in Fig. 3 the obtained life time is about 70 fs. The minimum *χ*^2^ value of the fit, however, is higher, i.e., worse, than for the screened Coulomb potentials. This indicates that the *ab initio* potential does not well describe the slowing down in the crystal. This may be due to some crucial approximations within the *ab initio* calculations, e.g., with respect to the exchange energy, that have to be made. Further approaches will be made in order to improve the potential.

## 8. Conclusion

Crystal-GRID measurements have been performed with ZnS single crystals. For the first time, the theoretically predicted asymmetric line shape could be verified experimentally. At the actual state of data evaluation it seems to be clear that the lifetime of the 3221 keV level in ^33^S is about 60 fs. This value is well above the previously reported value. Therefore the line shapes are less structured than predicted.

Due to the reduced information that can be extracted from the line shapes, a third ZnS measurement has been proposed. It should be performed in the 〈100〉 orientation. With the additional results it should be possible to validate interatomic potentials even though, due to the “long” lifetime, the transition is not optimal for Crystal-GRID studies.

The first *ab initio* results do not yet yield sufficiently good results. Nevertheless the simultaneous experimental and theoretical approaches allow a direct comparison of the results, leading to an increased reliability of the obtained potentials.
